# Post-Radiotherapy Dysphagia in Head and Neck Cancer: Current Management by Speech-Language Pathologists

**DOI:** 10.1007/s11864-024-01198-0

**Published:** 2024-05-01

**Authors:** Roganie Govender, Nicky Gilbody, Gavriella Simson, Rhiannon Haag, Ceri Robertson, Emma Stuart

**Affiliations:** 1 Head & Neck Academic Centre& UCL Division of Surgery & Interventional ScienceGround Floor Central, University College London Hospital, 250 Euston Road, London, NW1 2PQ UK; 2 North Middlesex University Hospital NHS Trust, London, UK; 3 Imperial College Healthcare NHS Trust, London, UK; 4 North East London NHS Foundation Trust, London, UK; 5 Sheffield Teaching Hospitals NHS Foundation Trust, Sheffield, UK; 6 Barking, Havering and Redbridge University Hospitals NHS Trust, Romford, UK

**Keywords:** Dysphagia, Head and neck cancer, Speech-language pathology, Swallow rehabilitation, Radiotherapy, Late-effects dysphagia

## Abstract

Dysphagia, difficulty in eating and drinking, remains the most common side effect of radiotherapy treatment for head and neck cancer (HNC) with devastating consequences for function and quality of life (QOL). Over the past decade, 5-year survival has improved due to multiple factors including treatment advances, reduction in smoking, introduction of the human papillomavirus (HPV) vaccine and more favourable prognosis of HPV-related cancers. Increased prevalence of HPV-positive disease, which tends to affect younger individuals, has led to an elevated number of people living for longer with the sequelae of cancer and its treatment. Symptoms are compounded by late effects of radiotherapy which may lead to worsening of dysphagia for some long-term survivors or new-onset dysphagia for others. Speech-language pathology (SLP) input remains core to the assessment and management of dysphagia following HNC treatment. In this article, we present current SLP management of dysphagia post-radiotherapy. We discuss conventional treatment approaches, the emergence of therapy adjuncts and current service delivery models. The impact of adherence on therapy outcomes is highlighted. Despite treatment advancements, patients continue to present with dysphagia which is resistant to existing intervention approaches. There is wide variation in treatment programmes, with a paucity of evidence to support optimal type, timing and intensity of treatment. We discuss the need for further research, including exploration of the impact of radiotherapy on the central nervous system (CNS), the link between sarcopenia and radiotherapy-induced dysphagia and the benefits of visual biofeedback in rehabilitation.

## Introduction

Dysphagia is an expected and prevalent issue experienced by patients undergoing radiotherapy or chemoradiotherapy for HNC. Symptoms of dysphagia including food sticking in the throat, coughing, and increased effort while swallowing food affect a third of patients before treatment due to the presence of the tumour. During radiotherapy and in the post-acute period, almost all patients report some level of dysphagia, and 6 months after treatment completion, as many as 50% of patients continue to experience symptoms of dysphagia [1]. Furthermore, it is now acknowledged that new-onset dysphagia often referred to as “late-effects” can manifest many years after treatment [2]. This delayed dysphagia is attributed to long-term tissue fibrosis, nerve damage and vascular impairment which collectively hinder the normal functioning of swallowing musculature [3•].

Beyond the evident issues related to reduced muscle movement impacting swallowing biomechanics, post-radiation dysphagia encompasses a broader range of contributing factors. These factors include taste (dysgeusia), dry mouth (xerostomia) and reduced jaw movement (trismus) all of which further influence the experience of eating and drinking. The consequences of a dysphagia diagnosis are substantial, exerting a notable impact on the QOL of survivors of HNC [1]. Additionally, it places a significant financial burden on healthcare services, potentially increasing costs by up to 40% for inpatients with dysphagia [4]. For patients with HNC, particularly those diagnosed and treated at a younger age, dysphagia can compromise their ability to return to work and social networks [5]. However, as emphasised earlier, dysphagia in this population is expected, often predictable and potentially amenable to interventions by SLPs.

In this review article, we focus on current SLP management of dysphagia for the post-radiotherapy population. We discuss conventional swallow rehabilitation, therapy adjuncts, biofeedback, recommendations for intensity and timing of intervention and service delivery models. Developing areas including the impact of radiotherapy on the CNS and the association between sarcopenia and post-radiotherapy dysphagia are explored with emphasis on the clinical implications.

## SLP management of dysphagia

Management of dysphagia is reliant on full and accurate assessment. Assessment of dysphagia in the HNC population typically consists of clinical and instrumental assessments in combination with patient-reported outcome measures (PROMs). Specific measures are outlined comprehensively in scientific literature [2, 6, 7]. Dynamic instrumental assessment of swallowing is considered gold standard. This includes videofluoroscopic swallow studies (VFSS) and fibreoptic endoscopic evaluation of swallowing (FEES), for which standardised tools have been developed to calculate swallow safety and efficiency [8, 9], representing the presence and degree of aspiration and post-swallow residue, respectively. Comprehensive assessment is essential in determining targeted clinical management that aligns with patient priorities. Below, we discuss SLP approaches to dysphagia management post-radiotherapy.

### Conventional swallow rehabilitation

Conventional swallow rehabilitation consists of compensatory techniques and direct exercises. Compensatory techniques aim to mitigate against dysphagia symptoms but do not facilitate a sustained change to swallow physiology. Such techniques include the use of modified diet and fluids and/or postural adjustment/s [10]. By contrast, direct exercises aim to strengthen swallow musculature, thereby improving swallow physiology [10].

#### Compensatory techniques

Diet and fluid modification is frequently used in the clinical setting to manage dysphagia symptoms and radiotherapy toxicities. While thickened fluids have historically been used in clinical practice to manage aspiration, there remains limited high-quality evidence to support their use in reducing incidence of aspiration pneumonia [11]. Research has, however, demonstrated that use of thickened fluids may result in reduced fluid intake thereby leading to dehydration [12]. Consequently, clinicians may avoid the use of thickened fluids in the HNC population outside agreed use for comfort. A free water protocol in the presence of aspiration may be recommended for swallow maintenance and QOL. Recent studies have demonstrated an absence of negative clinical indicators associated with this approach [13].

Postural adjustments (see Table 1) may be used alongside, or independently of, diet and fluid modification. The most used are briefly summarised in Table 1.

**Table 1:** Postural adjustments [10]

Posture	Rationale
Neck extension	Facilitates bolus flow from the oral to the pharyngeal cavity in the context of lingual weakness
Head turn	Compensates for unilateral pharyngeal and/or laryngeal weakness. Rotation to the side of impairment facilitates airway closure during the swallow and redirects the bolus towards the unimpaired side, thus aiding flow through the pharynx
Chin tuck	Facilitates airway closure in the context of delayed swallow initiation or reduced base of tongue retraction

#### Direct exercises

Direct exercises target specific aspects of swallow biomechanics. Given that radiotherapy affects multiple swallow parameters, rehabilitation programmes typically consist of multiple exercises [14–16]. The two main foci are swallow safety (reducing the presence of aspiration) and swallow efficiency (reducing the presence of post-swallow residue). Rehabilitation programmes may also include oromotor exercises targeting strength and range of movement of specific anatomical regions such as the lips and tongue [17]. Systematic reviews have demonstrated that swallow exercises are beneficial to swallow physiology and function following chemo/radiotherapy for HNC; however, further research is required to guide specific intervention protocols [17, 18].

##### Swallow safety

Structures critical to airway protection are frequently affected by radiotherapy with consequences for swallow safety. Multiple exercises broadly target swallow safety including the Shaker manoeuvre, chin tuck against resistance (CTAR), Mendelsohn manoeuvre, supraglottic swallow, vocal glides and singing therapy [14–16, 19, 20]. Typical SLP practice involves selection of the most appropriate targeted exercises to facilitate patient engagement. For example, a systematic review has demonstrated that CTAR is equally as effective as the Shaker in terms of improvements in swallow physiology and results in less physical strain on the patient [15]. Consequently, selection of the CTAR may yield similar clinical benefits and lead to greater adherence.

##### Swallow efficiency

Reduced base of tongue retraction and pharyngeal drive are hallmark features of post-radiotherapy dysphagia resulting in reduced swallow efficiency and consequent pharyngeal residue. Exercises targeting base of tongue retraction and pharyngeal drive include the Masako manoeuvre and effortful swallow, respectively [21, 22].

### Therapeutic devices

**Fig. 1:**
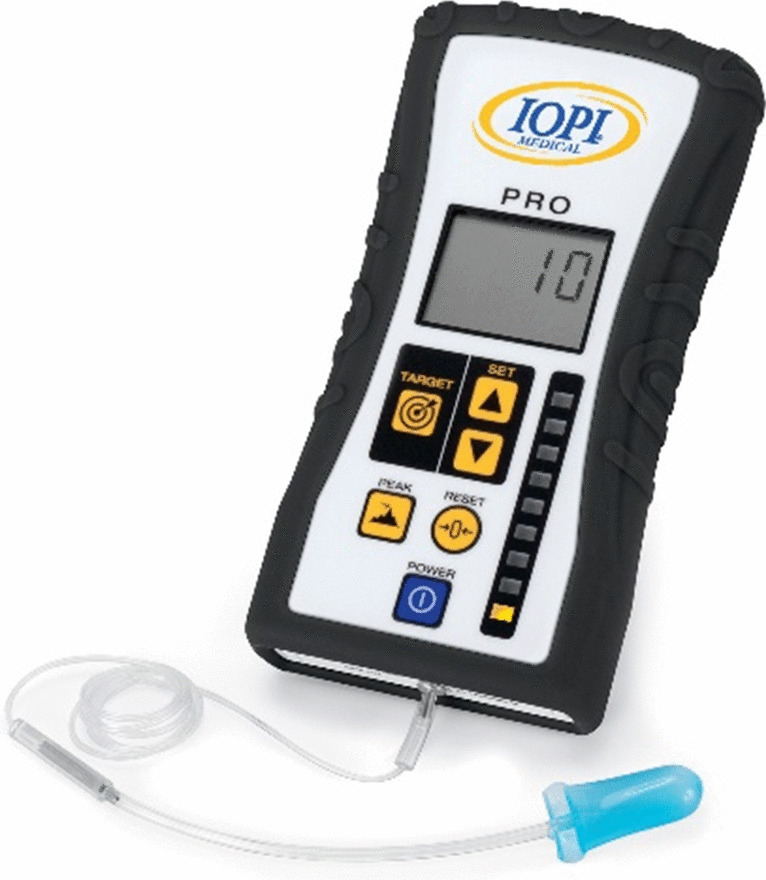
IOPI® device. Printed with permission from IOPI Medical LLC.

In addition to conventional swallow exercises, therapy devices can support rehabilitation. The Iowa Oral Performance Instrument (IOPI®) trainer (see Figure 1) and Expiratory Muscle Strength Training (EMST) target tongue and expiratory muscle strength, respectively [23].

The IOPI® is a small portable device containing an attachment to an air-filled balloon. The patient is instructed to squeeze the bulb between the tongue and the palate. Tongue pressure is measured, with visual biofeedback provided on a digital display when the patient reaches their target [24]. While HNC-specific trials are limited, IOPI® has been shown to be beneficial in the management of dysphagia following stroke and acquired brain injury [25]. Results of a prospective case series study of individuals presenting with chronic radiation-induced dysphagia demonstrated a significant improvement in tongue strength following treatment; however, improvements in oral intake, patient-reported health status and QOL did not reach statistical significance [25].

The EMST is a handheld portable device which targets airway protection. The patient exhales forcefully into a one-way spring-loaded valve. The valve is tightened incrementally, increasing the amount of resistance required for the expiratory and suprahyoid muscles to overcome [23]. EMST targets airway protection via two mechanisms:

1. Strengthening the expiratory muscles facilitating improved cough strength and therefore promoting clearance of aspiration from the lower airway [26]
2. Strengthening the suprahyoid muscles, facilitating improved airway closure

There is evidence to support the use of EMST in the management of dysphagia related to neurogenic pathologies including Parkinson’s disease, amyotrophic lateral sclerosis and stroke [23]. A small retrospective case series study assessing the efficacy of EMST in the HNC population demonstrated significant improvements in maximum expiratory pressure and a reduction in aspiration following an 8-week course of EMST. Changes to QOL, post-swallow residue and diet did not reach statistical significance [23].

Early results of studies assessing the efficacy of IOPI® and EMST in the HNC population demonstrate positive outcomes. At the time that this article was written, further trials were underway to assess the efficacy of IOPI® and EMST in the target population in both the prehabilitation and rehabilitation contexts [24, 27].

Surface electromyography (sEMG) is a therapy adjunct described in the literature but not routinely used in clinical practice [28, 29]. Electrodes placed in the submental region provide information regarding muscle activity including performance of a swallow or swallowing exercise [29]. Constantinescu et al. [30] used sEMG alongside exercises specifically selected for their increased physiological load (effortful swallow and Mendelsohn manoeuvre) to monitor exercise performance and provide visual biofeedback. Outcomes revealed improvements in dysphagia-related QOL following a 6-week programme.

The TheraBite® Jaw Motion Rehabilitation System™ (Atos Medical) is a patient-controlled device which targets trismus. The device has two mouth pieces which are placed between the upper and lower jaws. The patient squeezes the handles which passively opens the jaw [31]. While this does not target swallow musculature per se, optimal jaw opening facilitates oral intake. A study assessing the feasibility and cost-effectiveness of the TheraBite® is currently underway [32].

A device to support CTAR has been developed by Atos Medical. This handheld device contains two bars with one bar placed submentally and the other on the sternum. Resistance is increased as required. This device has been trialled in stroke and frailty populations [33, 34], and a preliminary study including CTAR, jaw opening against resistance exercises and effortful swallow yielded promising results with HNC survivors [35]. A larger scale study exploring the use of this device in conjunction with effortful swallow for the management of chronic radiation-induced dysphagia is currently underway [36].

### Biofeedback

As discussed briefly in relation to IOPI® and sEMG, therapy devices have a further anticipated benefit with regard to their capacity to provide visual biofeedback. Due to alterations in sensation following radiotherapy, patient perceptions of swallowing dysfunction may not correlate with clinician-reported measures [37]. As a result, patients may be less inclined to engage in direct exercises. The integration of visual biofeedback into rehabilitation may facilitate behaviour change and adherence to therapy programmes. Visual biofeedback may also enable error-based learning to develop or re-learn a skill [38]. While preliminary research involving healthy older adults suggests improved outcomes when combining visualisation techniques with traditional therapy [39], studies pertaining specifically to HNC are comparatively few.

To date, FEES is used predominantly as an assessment tool; however, extending its use to rehabilitation may be beneficial. The use of real-time visual biofeedback during FEES may facilitate teaching of swallow exercises such that the patient can practice and receive live feedback regarding the accuracy of performance [38]. FEES does not use radiation and can therefore be completed with increased frequency when compared with VFSS [40].

### Timing and intensity of treatment

Consideration of intensity and timing of treatment is critical to the planning of swallow rehabilitation [41]. An overall benefit to swallow rehabilitation following curative chemoradiotherapy has been suggested, regardless of the timing of intervention [17]. However, although the majority of SLPs recommend post-treatment maintenance programmes [42], there is a paucity of evidence defining optimal timing and intensity of intervention [7, 17, 43].

There has been a recent increase in high-intensity rehabilitation programmes which are based on exercise physiology and neuroplasticity principles [44]. These programmes adopt a functional approach to rehabilitation targeting the entire swallow process and increasing the load on swallow musculature. Interventions are offered over a 3–10-week period and include progressive bolus loads with increasing volume and viscosity [44]. The McNeill Dysphagia Therapy Programme advocates 100 functional swallows per day [45] or 60 min of once daily practice, 5 days per week [44]. Research has demonstrated that this approach results in improved QOL and swallow function as demonstrated on VFSS [44]. However, adherence to high-intensity programmes is limited with the time commitment likely to be a contributing factor to reduced engagement [6, 41].

At the time that this article was written, the PRO-ACTIVE trial was in progress. This trial aims to assess swallow outcomes of a proactive oral intake maintenance programme when compared with reactive interventions. Factors including timing and intensity are explored [46].

### Adherence

Adherence to rehabilitation recommendations remains a barrier to achieving optimal swallow outcomes. Research has revealed that only 13–14% of patients practice swallowing exercises as recommended [47]. This is despite a demonstrated association between adherence to recommendations and improved swallowing outcomes [48]. The engagement of the healthcare system with individuals and the degree to which an individual’s values, needs and social context are considered may be contributing factors to poor adherence [48, 49]. Furthermore, an individual’s ability to participate in rehabilitation may vary according to their position along the cancer-care continuum, in line with physical and emotional responses to the disease and its treatment [50]. Remedying this requires co-creation of treatment plans involving the individual, their family, and the multidisciplinary team (MDT). Goals must be meaningful and personalised according to swallow function, psychosocial function, age, sex, comorbidities, primary aetiology, motivation and physical fitness [48] if intention is to translate into behaviour [51].

There is increasing evidence to support the integration of behaviour change principles into rehabilitation programmes. A systematic review [52] identified key behaviour change techniques which occur more frequently in effective swallow interventions and promote adherence. SLPs report improved patient understanding of the rehabilitation process when behaviour change principles are implemented; however, evidence for the impact on patients is currently still being accrued via ongoing clinical trials [53, 54].

Finally, there is a developing evidence base for the inclusion of cognitive behavioural therapy (CBT) principles into rehabilitation. Research suggests that this approach results in improved QOL and increased confidence and adjustment [43]. It is worth noting that the inclusion of a full CBT programme into swallow rehabilitation may be cost and resource prohibitive for most services.

### Service delivery

The COVID-19 pandemic has accelerated the evolution of service delivery models through the increased use of virtual modalities to supplement essential in-person appointments. Remote services are more commonplace, particularly in rural communities or remote geographical areas, allowing for improved access to care alongside service and cost efficiencies [55–57].

Although study numbers were small, findings of a systematic review [58] suggest that telemedicine may be an effective tool in symptom management in the HNC population. Its use may improve adherence to rehabilitation protocols when compared with self-directed rehabilitation used in isolation [58]. Both assessment and intervention components of SLP dysphagia management can take place virtually. Remote dysphagia assessment including clinical evaluation and VFSS has been considered safe, valid and reliable [59]. Recent studies indicate that intervention can be offered successfully via telehealth as synchronous (live) sessions or using “store and forward” technologies without compromising learning and with a high degree of patient satisfaction [60, 61]. Telemedicine can also form an integral part of MDT discussion, leading to timely interprofessional decisions around dysphagia intervention across healthcare services [62].

Technologically enhanced home-based swallowing therapies use mobile systems, apps, or interactive websites in combination with traditional therapy techniques. These approaches address acknowledged low adherence rates in home programmes for this cohort and allow clinicians to remotely monitor progress [63, 64]. Similarly, smartphone applications and interactive websites have been developed to support prehabilitation and rehabilitation packages [65–67]. These are designed to support service users through use of video, audio and photographic images to demonstrate strategies, diet modification ideas and therapeutic exercises. The online platforms or apps encourage users to enter data, allowing progress to be monitored by SLPs.

These approaches have demonstrated clinically equivalent outcomes when compared with traditional face-to-face therapy in isolation. In addition, they have contributed to improved adherence to swallowing and trismus exercises, which has been associated with improved patient-reported QOL [68, 69]. Challenges with new technologies include accessibility and navigability for different populations, regulation and acceptance by clinicians and patients [62]. However, the shift towards more flexible service models supports the changing population of HNC survivors, with structured support facilitating self-management and ownership of health conditions [70, 71].

### Palliation

In palliative and end-of-life care, SLPs work closely with the wider multidisciplinary team (MDT) to consider intervention approaches, with recognition of confounding preexisting difficulties when treating disease recurrence [7]. SLPs, alongside other healthcare professionals, have a responsibility for ongoing advocacy for this patient cohort through palliation [72]. Input focuses on individualised, person-centred optimisation of communication, swallow function and QOL, alongside supporting psychosocial and practical needs and symptom management in the context of declining health [73]. Intervention may include any/all of the elements previously discussed, alongside patient and family education. SLPs also address eating and drinking with acknowledged risks, assessing and quantifying the risks and benefits of continued oral intake, adding contextual information on disease process and progress and delivering accessible information to facilitate informed choice [74]. This requires in-depth understanding and negotiation around ethical issues, for example appropriacy of nil by mouth status at end of life and consideration of oral intake for comfort or pleasure [74].

## Developing areas

Historically, swallow rehabilitation has targeted the peripheral nervous system (PNS), aiming to strengthen swallow musculature. Emerging research has demonstrated that late-effects dysphagia may affect both the PNS and central nervous system (CNS) [75, 76, 77•]. Inclusion of the medulla oblongata in radiotherapy fields has been found to be significantly associated with dysphagia at 1 year post-treatment [77•]. The impact of radiotherapy on both the PNS and CNS may explain airway protection impairments [78] which have been associated with aspiration in HNC survivors [26]. This understanding has led to new therapeutic approaches, adding skill training to traditional strengthening approaches [79]. Fullerton et al. [80] studied changes in reflexive and volitional cough parameters after radiotherapy, postulating that cortical plasticity might be used to overcome fibrosis-linked impairments. However, while cough skill training approaches have proven successful in neurological conditions [81, 82], studies in HNC are lacking. Fullerton et al. [80] also suggested that sensory deterioration precedes motor impairment and therefore this trajectory may be inhibited by increasing conscious control of swallowing and using sensory stimuli at an early stage. Research in this area is ongoing. HIT-CRAD [36] is a multicentre randomised trial currently exploring intensive treatment of chronic radiation-associated dysphagia in HNC survivors, with the aim of investigating the role of cortical plasticity in swallow rehabilitation. The results from this trial may help inform future intervention.

We have previously discussed the benefits of visual biofeedback in facilitating swallow rehabilitation, particularly with regard to skill training. Further biofeedback tools include high-resolution manometry [83] and ultrasound [84]. Blyth et al. [85] reported a single-case experimental study of ultrasound-guided rehabilitation after partial glossectomy; however, the evidence supporting the use of ultrasound as a biofeedback tool post-radiotherapy is limited. Early research into the use of high-resolution manometry includes a case study and a case series study which included one participant with HNC [86, 87]. Further research in this area may benefit clinical practice.

Sarcopenia has recently been associated with radiation-induced dysphagia [88•]. Adequate assessment of sarcopenic dysphagia is critical to the development of realistic swallow rehabilitation goals. Preliminary research has demonstrated the role of ultrasound in the assessment of sarcopenic dysphagia [89–91]. Rehabilitation studies into sarcopenic dysphagia in HNC are embryonic but have demonstrated encouraging results. A recent feasibility trial assessing the benefits of a strength and skill-based dysphagia rehabilitation programme included two participants with HNC who reported positive change [92]. A case study completed by Hashida et al. [93] demonstrated improvements in sarcopenic dysphagia post-glossectomy following physical therapy, nutritional intervention and dysphagia therapy. However, further research is required to fully explore the efficacy of swallow rehabilitation for sarcopenic dysphagia post-radiotherapy.

There is emerging research into use of technologically enhanced interventions, and while further work is needed to support its efficacy in clinical practice, use of artificial intelligence may facilitate more targeted decisions around therapy [94, 95].

## Conclusion

While 5-year survival following treatment for HNC has improved, treatment advances and increased prevalence of HPV-associated disease have led to an increase in the number of people living for longer with the effects of cancer and its treatment. This has led to an increased incidence of chronic post-radiotherapy dysphagia. We have outlined the current SLP management of post-radiotherapy dysphagia and discussed gaps in the evidence base. Key research priorities include criteria for selection of swallowing exercises and therapy adjuncts, establishment of rehabilitation protocols with specifications around timing and intensity and best use of service delivery models. There is a clear need for well-designed trials with adequate sample sizes and inclusion of validated baseline and endpoint outcome measures to inform clinical decisions regarding swallow rehabilitation. Integration of device-driven, functional skill-based activity and strengthening exercises with individualised swallow programmes may yield the best results within a holistic model of care [6, 36]. Critical to the delivery of targeted swallow rehabilitation is an improved understanding of the impact of radiotherapy on the CNS and the association between radiation-induced dysphagia and sarcopenia. There remains a need for an improved understanding of preexisting comorbidities on post-radiation dysphagia. SLPs remain key members of the multidisciplinary team, providing essential advocacy and support to people with dysphagia as a result of HNC or its treatment, from point of diagnosis through to palliation.
